# Mitochondrial DNA leakage exacerbates odontoblast inflammation through gasdermin D-mediated pyroptosis

**DOI:** 10.1038/s41420-021-00770-z

**Published:** 2021-12-09

**Authors:** Yi-Fei Zhang, Lu Zhou, Han-Qing Mao, Fu-Hua Yang, Zhi Chen, Lu Zhang

**Affiliations:** 1 The State Key Laboratory Breeding Base of Basic Science of Stomatology (Hubei-MOST) & Key Laboratory of Oral Biomedicine Ministry of Education, School & Hospital of Stomatology, Wuhan University, Wuhan, China; 2 Department of Endodontics, School and Hospital of Stomatology, Wuhan University, Wuhan, China

**Keywords:** Cell death, Cell death and immune response

## Abstract

Alleviating odontoblast inflammation is crucial to control the progression of pulpitis. Mitochondrial DNA (mtDNA) is a vital driver of inflammation when it leaks from mitochondria of inflamed odontoblasts into the cytosol. Bacteria-induced inflammation leads to a novel type of cell death named pyroptosis. The canonical pyroptosis is a gasdermin (GSDM)-dependent cytolytic programmed cell death characterized by cell swelling and pore formation in the plasma membrane. To date, whether odontoblast cytosolic mtDNA regulates dental pulp inflammation through the canonical pyroptosis pathway remains to be elucidated. In this study, high gasdermin D (GSDMD) expression was detected in human pulpitis. We found that LPS stimulation of mDPC6T cells promoted BAX translocation from the cytosol to the mitochondrial membrane, leading to mtDNA release. Moreover, overexpression of isolated mtDNA induced death in a large number of mDPC6T cells, which had the typical appearance of pyroptotic cells. Secretion of the inflammatory cytokines CXCL10 and IFN-β was also induced by mtDNA. These results suggest that cytosolic mtDNA participates in the regulation of odontoblast inflammation through GSDMD-mediated pyroptosis in vitro. Interestingly, after overexpression of mtDNA, the expression of inflammatory cytokines CXCL10 and IFN-β was increased and not decreased in GSDMD knockdown mDPC6T cells. We further proposed a novel model in which STING-dependent inflammation in odontoblast-like cell is a compensatory mechanism to control GSDMD-mediated pyroptosis, jointly promoting the immune inflammatory response of odontoblasts. Collectively, these findings provide the first demonstration of the role of the mtDNA-GSDMD-STING in controlling odontoblast inflammation and a detailed description of the underlying interconnected relationship.

## Introduction

According to Global Burden of Diseases, Injuries, and Risk Factors Study 2016 data, global incidence of dental caries was the second [1]. When the hard tissue of the tooth is damaged due to caries, bacteria can enter through the defect and infect the pulp tissue, causing pulp inflammation [2]. Pulpitis is a common oral inflammatory disease in the clinic and seriously affects people’s daily lives. Odontoblasts in dental pulp tissue are the first line of defense against bacteria [3]. Alleviating inflammation of odontoblasts is critical to control the progression of pulpitis. Inflammation is a part of the customary protective immune response and it promotes recruitment of immune cells to the damage site and eliminates injurious pathogens. Mitochondrial damage can cause the release of mitochondrial ROS, mitochondrial DNA, Cytochrome c and other substances [4, 5] and play an important role in the pathogenesis of pulpitis. Mitochondrial DNA (mtDNA) is the genetic material of mitochondria and primarily encodes proteins for oxidative phosphorylation, which provides energy for various life activities [6].

mtDNA is an essential activator of inflammation when it leaks from damaged mitochondria after endogenous or exogenous stimulation [7]. Mild and short-term stimulation causes partial degradation of mitochondria and leads to the occurrence of mitophagy [8]. Strong and continuous stimulation can cause mtDNA leakage and induce various types of programmed or unprogrammed cell death, thereby triggering an immune response [9]. Cytosolic mtDNA participates in inflammation progression in various diseases, such as rheumatoid arthritis [10], lupus and atherogenesis [7]. Our previous studies indicate that lipopolysaccharide (LPS) induces odontoblast mitochondrial dysfunction and mtDNA leakage to activate robust inflammation [8, 11]. Cytosolic mtDNA can directly activate various pattern recognition receptors (PRRs) to cause inflammation, such as TLR9 and inflammasomes [6]. Bacteria-induced inflammation is a novel type of cell death named pyroptosis, which is an executioner that operates downstream of inflammasome activation [12]. However, the mechanism by which cytosolic mtDNA participates in odontoblast inflammation is unclear.

Pyroptosis is a gasdermin (GSDM)-dependent cytolytic programmed cell death characterized by cell swelling and pore formation in the plasma membrane. Eventually, the cell membrane dissolves and a large amount of cell contents is released [13], thereby triggering an immune response and inflammation in the tissue and resulting in local or even systemic inflammation and immune pathological damage [10, 14]. The canonical pyroptosis occurs through different inflammasomes (such as NLRP3 and AIM2) and the gasdermin D (GSDMD) pathway. After LPS stimulation induces activation of inflammasomes and caspase-1, GSDMD, which is the key molecule in pyroptosis, is cleaved into two fragments: the C-terminal domain and the N-terminal domain. Then, the cleaved N-terminal domain anchors to the cell membrane, leading to pore formation and causing leakage of cell contents, such as IL-1β [13, 15]. In addition to robust pyroptosis induction, GSDMD can regulate the STING pathway through ion channels and participate in the progression of inflammation [16, 17]. Furthermore, studies have shown that released mtDNA can activate the NLRP3 inflammasome and inflammatory caspase-1, leading to release of IL-1β from macrophages [9, 18]. However, the mechanism of pyroptosis in pulpitis remains curious. Nonetheless, whether cytosolic mtDNA in odontoblasts regulates dental pulp inflammation through the canonical pyroptosis pathway remains to be elucidated.

In the present study, we examined whether LPS-induced mtDNA leakage participates in regulation of odontoblast inflammation through GSDMD-mediated pyroptosis in vitro. The pyroptosis pathway and the STING pathway cooperate with each other to jointly promote the immune inflammatory response of odontoblasts. This study reveals a new mechanism of cytosolic mtDNA-mediated inflammation of odontoblasts and is expected to provide new therapeutic targets for controlling pulpitis progression.

## Materials and methods

### Human dental pulp samples

Human dental pulp tissue was collected as described above [11, 19] and separated into two groups: 16 healthy teeth and 16 pulpitis teeth. The healthy group included no history of dental pulp disease and no oral diseases, such as dental caries. The pulpitis group included prolonged spontaneous pain and a history of severe pain. These procedures were conducted in accordance with the guidelines of the National Institutes of Health on the use of human tissue and were approved by the Institutional Ethics Committee of Wuhan University (2019 LUNSHENZI(A48)).

### Immunohistochemistry staining

All human pulp samples from the healthy and pulpitis groups were demineralized in 10% EDTA for 3 months, dehydrated and rooted in paraffin to obtain 6 μm paraffin tissue sections. The tissue sections were incubated with primary antibody against cleaved GSDMD (#36425; Cell Signaling Technology, USA) for immunohistochemistry staining overnight at 4 °C. Incubation with secondary antibody and the subsequent colorization were performed as described in our previous research [20].

### Cell culture and LPS treatment

mDPC6T is a pre-odontoblast cell line that highly expresses DMP1, DSP and other odontoblast-related markers and has a phenotype similar to that of terminally differentiated odontoblasts [3]. The mDPC6T cell line was established and is maintained by our team. mDPC6T cells were cultured in Dulbecco’s modified Eagle medium (DMEM, HyClone, USA) containing 10% fetal bovine serum (FBS, Gibco, USA) at 37 °C in a humidified air atmosphere containing 5% CO_2_. The cells were treated with the indicated lipopolysaccharide (LPS, Sigma, USA) concentration at the indicated time, and the mitochondrial DNA replication inhibitor Ethidium Bromide (EtBr, Thermo Fisher Scientific, USA) was dissolved in DMEM to produce a final concentration of 1 μg/ml.

### Cytosolic mtDNA copy number analysis

Mitochondria were isolated from mDPC6T cells using a mitochondrial isolation kit (BioVision, USA). After the mitochondria were discarded, the cytoplasm was prepared to isolate cytosolic mtDNA using a genomic DNA miniprep kit (Axygen, USA). PCR quantification was then carried out to analyze mitochondrial DNA and nuclear DNA in the cytoplasm. Mitochondrial DNA was marked by tRNA-Leu^UUR^, while nuclear DNA was characterized by β-microglobulin genes. The ratio of mtDNA to nDNA in the cytoplasm represented the copy number of cytosolic mtDNA.

### Mitochondrial DNA isolation and transfection

Mitochondrial DNA isolation kit (BioVision, USA) was used to isolate mitochondrial DNA from mDPC6T cells. mDPC6T cells were transfected with 1 μg/well mtDNA for 24 h using Lipofectamine 3000 (Thermo Fisher Scientific, USA).

### Small interfering RNA transfection

mDPC6T cells were transfected with GSDMD-targeted small interfering RNA (GenePharma China, Shanghai). The cells were transfected twice using Lipofectamine 3000 (Invitrogen, USA) at an interval of 48 h. Total RNA and protein were collected after the second transfection with mtDNA for 24 h to verify the effect of protein knockdown.

### RNA quantification (qPCR)

Total RNA was isolated from a pre-odontoblast cell line using a total RNA miniprep kit (Axygen, USA). A Reverse Transcription System (Vazyme, China) was used to perform cDNA synthesis, and a Bio-Rad-CFX96 real-time PCR system was used to perform qPCR. The primers are listed in the appendix.

### Immunofluorescence and confocal laser scanning microscopy

Cells were grown on confocal dishes and then treated with 20 µg/mL LPS for 24 h. mDPC6T cells were incubated with 200 mM MitoTracker (Invitrogen, USA) for 15 min and then fixed with paraformaldehyde, permeabilized with 3% Triton X-100 and blocked with 10% bovine serum albumin for 1 h. Cells were incubated with primary antibody at 4 °C overnight, and the primary antibody information is listed in the appendix tables. Images were acquired using confocal microscopy (Olympus, Japan).

### Western blotting

Total protein was extracted via RIPA buffer (Beyotime, China) with proteinase inhibitor and phosphatase inhibitor (MCE). Mitochondrial and cytoplasmic proteins were isolated and extracted with a mitochondrial isolation kit (Beyotime, China). Western blotting was performed as previously described [19]. Relevant information on the primary antibodies used is listed in the appendix tables.

### Flow cytometry

After treatment with LPS, cells were incubated with 5 μM MitoSOX Red Dye for 30 min, and the release of mitoSOX was analyzed via flow cytometry (Beckman, USA).

### LDH release assay

Cells were seeded into a 96-well plate and transfected with mtDNA for 24 h. An LDH Assay kit (Beyotime, China) was used to detect the release of LDH by the cells according to the manufacturer’s instructions.

### Statistical analyses

All data are presented as the means ± standard deviations obtained from three independent experiments. GraphPad Prism 8.0 was used to analyze all data. All experiments were repeated at least three times and the differences were analyzed by one-way ANOVA or the Student’s *t* test. *P* value of <0.05 was considered statistically significant.

## Results

### LPS-induced mitochondrial damage and mtDNA leakage via BAX

**Fig. 1:**
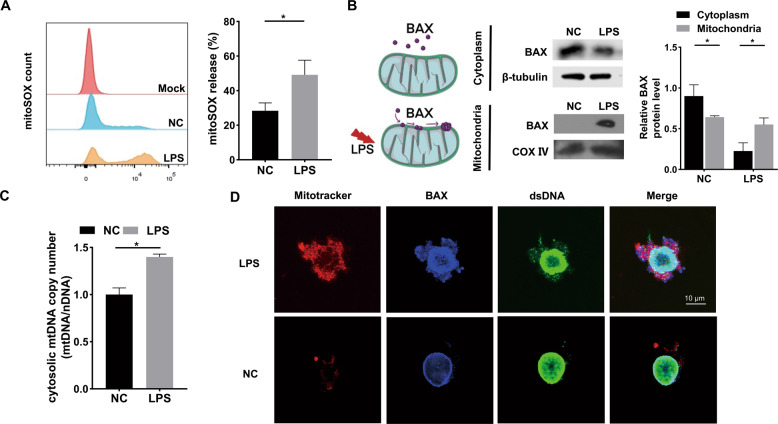
LPS-induced mitochondrial damage and mtDNA leakage into cytosol via BAX. **A** The release rate of mitoSOX in NC and LPS groups was measured by flow cytometry (*n* = 3). **B** The cytoplasmic and mitochondrial protein level of BAX was analyzed by Western blotting after LPS stimulation (20 μg/ml, 24 h). β-tubulin served as the loading control for cytoplasm. COX IV served as the loading control for mitochondria. **C** qPCR was performed to assess the cytosolic mtDNA copy number in mDPC6T cells after LPS treatment. **D** Confocal microscopy to detect the co-location of immunofluorescence labeled mitochondria, BAX and dsDNA after 24 h of LPS treatment. MitoTracker stained mitochondria (red). Anti-BAX antibodies stained BAX (blue). Anti-dsDNA antibodies stained double-stranded DNA (green). Scale bar: 10 μm. The data are the means ± standard deviations of 3 independent experiments (*p* < 0.05^*^, *p* < 0.01^**^, *p* < 0.001^***^).

To investigate the relationship between mitochondria and inflammation, we analyzed mitochondrial function and the release of mitoSOX after LPS stimulation of mDPC6T cells. The release of mitoSOX in the LPS-treated group significantly increased (Fig. 1A), indicating that LPS caused mitochondrial damage in mDPC6T cells. BAX is a cytoplasmic protein in the Bcl-2 family [21]. The collapse of membrane potential caused by mitochondrial damage can induce activation of BAX protein, which is then transferred to the mitochondrial outer membrane and makes it permeable, forming pores and causing release of mitochondrial contents, such mitochondrial DNA [21–23] (Fig. 1B). To confirm the role of BAX in mitochondrial damage in mDPC6T cells, we isolated mitochondria and cytoplasm separately. Compared with the control group, the expression of BAX in the LPS-stimulated group decreased in the cytoplasm but increased in the mitochondria (Fig. 1B). The findings showed that BAX was transferred from the cytoplasm to mitochondria after LPS treatment. Furthermore, we detected the enrichment of mtDNA in the odontoblast cytoplasm and observed that the copy number of cytoplasmic mtDNA was significantly increased after LPS treatment (Fig. 1C). Immunofluorescence showed corresponding results: BAX and dsDNA content in the cytoplasm increased and colocalized with mitochondria after LPS treatment (Fig. 1D). From the above results, we found that LPS caused BAX translocation from the cytoplasm into mitochondria and pore formation, leading to mtDNA leakage.

### GSDMD was elevated in human patients with pulpitis

**Fig. 2:**
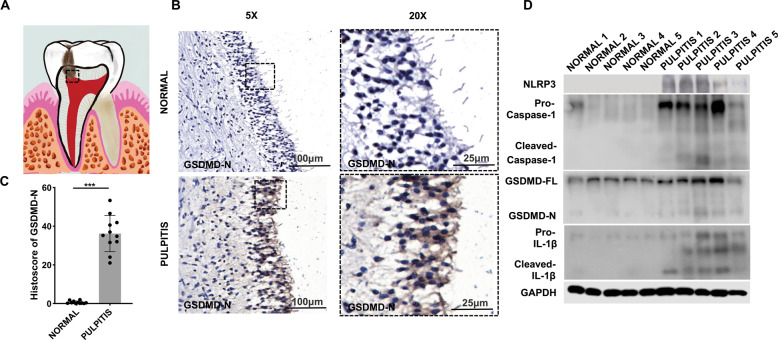
The expression level of GSDMD-N and pyroptosis-associated proteins was elevated in the inflamed dental pulp. **A** The schematic model of pulpitis. **B** Immunohistochemical (IHC) staining showed that the expression of GSDMD-N in normal tissue and pulpitis tissue. **C** Histoscores of GSDMD-N was determined from GSDMD-N expression level in normal tissue (*n* = 11) and pulpitis tissue (*n* = 11). **D** Western blotting was used to analyze the expression of pyroptosis-associated proteins in normal tissue and pulpitis tissue (*n* = 5). The data are the means ± standard deviations of three independent experiments (*p* < 0.05^*^, *p* < 0.01^**^, *p* < 0.001^***^).

Emerging evidence has shown that cytosolic mtDNA acts as a DAMP to activate NLRP3 or AIM2 inflammasomes [7, 23]. The NLRP3 inflammasome can then further activate GSDMD-mediated pyroptosis [12, 15]. We rationally hypothesized that mtDNA is biologically involved in inflammation via GSDMD-mediated pyroptosis. Consequently, the cleaved GSDMD expression was markedly upregulated in inflammatory odontoblast layer (Fig. 2A–C). Higher protein level of cleaved GSDMD in inflammatory dental pulp tissue was consistent with the immunohistochemical staining results (Fig. 2D). Meanwhile, in pulpitis tissue, the protein level of NLRP3, cleaved caspase-1 and cleaved IL-1β, which also play a key role in inflammation and pyroptosis, was significantly increased (Fig. 2D). These data demonstrate a relationship between GSDMD-mediated pyroptosis and pulpitis progression.

### Cytosolic mtDNA induced mDPC6T cell pyroptosis via GSDMD

**Fig. 3:**
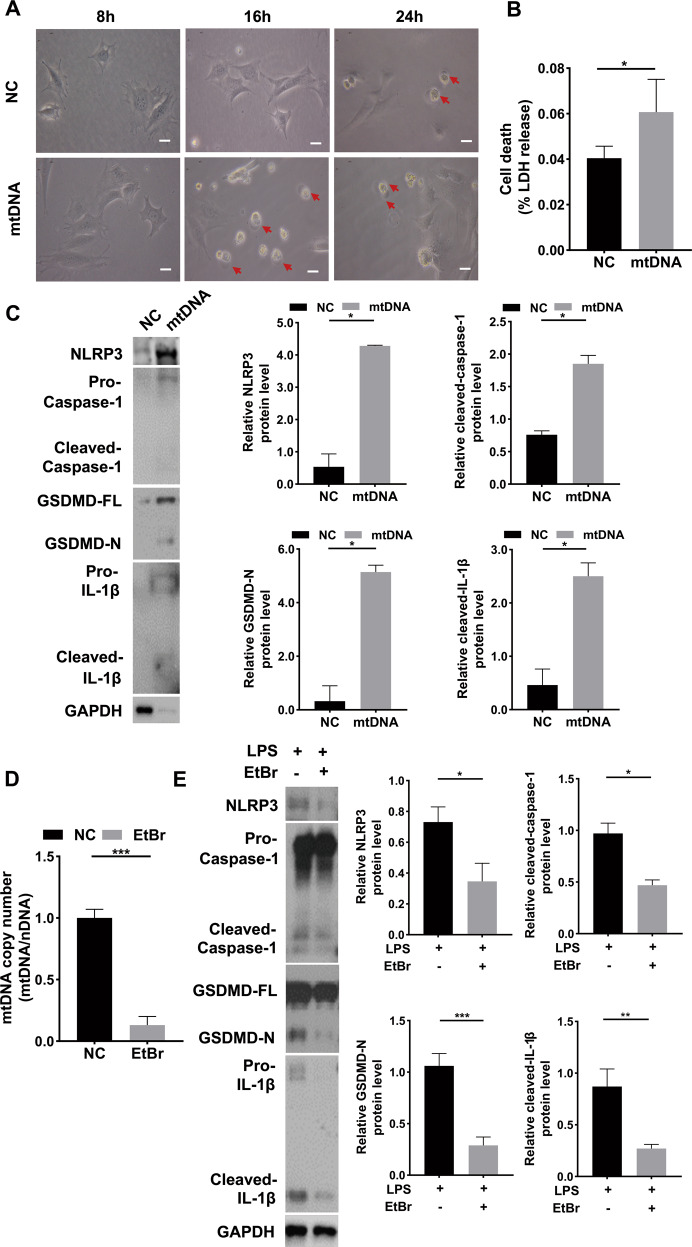
Cytosolic mtDNA induced mDPC6T pyroptosis. **A** Light microscopy examined the mDPC6T cells pyroptosis phenotype after transfected mtDNA (scale bar, 50μm). **B** LDH release assay was used to detect cell death after overexpression of mtDNA in mPDC6T cells (1 μg/well, 24 h). **C** The protein level of NLRP3, Caspase-1, GSDMD and IL-1β in mDPC6T cells was measured by western blotting after transfected mtDNA. **D** qPCR was performed to assess the mtDNA copy number in mDPC6T cells after EtBr treatment (1 μg/ml, 48 h). **E** The protein level of NLRP3, Caspase-1, GSDMD and IL-1β in mDPC6T cells after treatment with LPS and EtBr examined by immunoblotting. The data are the means ± standard deviations of 3 independent experiments (*p* < 0.05^*^, *p* < 0.01^**^, *p* < 0.001^***^).

To understand whether cytosolic mtDNA leads to mDPC6T cell pyroptosis. We isolated mtDNA from mDPC6T cells and overexpressed it in odontoblast-like cells. A large number of cells died after mtDNA overexpression and exhibited the typical appearance of pyroptotic cells under a microscope: the cells were swollen and round. Pyroptotic cells began to appear after 8 h of treatment, reached a peak at 16 h, and then decreased (Fig. 3A). After overexpression of mtDNA, the release level of LDH increased significantly, indicating that mtDNA can cause mDPC6T cell pyroptosis (Fig. 3B). Consistently, the protein level of NLRP3, cleaved caspase-1, cleaved GSDMD and cleaved IL-1β was remarkably increased after mtDNA overexpression (Fig. 3C). To further determine whether cytosolic mtDNA triggered pyroptosis in mDPC6T cells, mDPC6T cells were treated with mtDNA replication inhibitor Ethidium Bromide (EtBr) and the enrichment of mtDNA in the cytosolic compartment of mDPC6T cells was remarkably suppressed (Fig. 3D), confirming that mtDNA replication could be inhibited by EtBr. Consistently, the protein expression level of NLRP3, cleaved caspase-1, cleaved GSDMD and cleaved IL-1β was markedly decreased in the EtBr-stimulated group (Fig. 3E), indicating that inhibiting cytosolic mtDNA can decreased mDPC6T cells pyroptosis.

### Cytosolic mtDNA exacerbated inflammatory progression

**Fig. 4:**
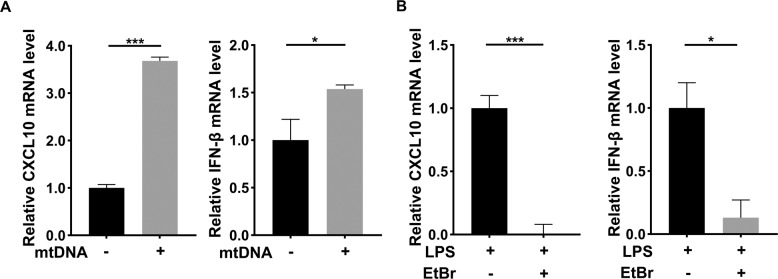
Cytosolic mtDNA exacerbated inflammatory progression. **A** The mRNA level of inflammatory cytokines, CXCL10 and IFN-β was detected by qPCR after overexpressing mtDNA (1 μg/well). **B** qPCR was performed to assess the CXCL10 and IFN-β mRNA level in mDPC6T cells after EtBr (1 μg/ml, 48 h) and LPS (20 μg/ml, 24 h) treatment. The data are the means ± standard deviations of three independent experiments (*p* < 0.05^*^, *p* < 0.01^**^, *p* < 0.001^***^).

To determine the effect of cytosolic mtDNA on odontoblast inflammatory progression, we detected the mRNA level of inflammatory cytokines after mtDNA overexpression. The result showed compared control group, inflammatory cytokines CXCL10 and IFN-β, was increased significantly in mtDNA overexpressing group (Fig. 4A), which demonstrated cytosolic mtDNA might exacerbated inflammatory progression. To confirm our hypothesis, we built a odontoblast inflammatory model. We assessed inflammatory cytokines after treated with mtDNA inhibitor EtBr in inflammatory environment. Consistent with the result of overexpressing mtDNA, the expression of inflammatory cytokines CXCL10 and IFN-β was suppressed after the treatment of EtBr (Fig. 4B). The results indicated that inhibiting mtDNA relieved inflammatory progression and decreased inflammatory cytokines secretion. In general, the results showed a similar tendency that mtDNA when it leaks from damaged mitochondria, as an essential activator of inflammation, exacerbated odontoblast inflammatory progression.

### Cytosolic mtDNA-mediated inflammation via the mtDNA-GSDMD-STING pathway

**Fig. 5:**
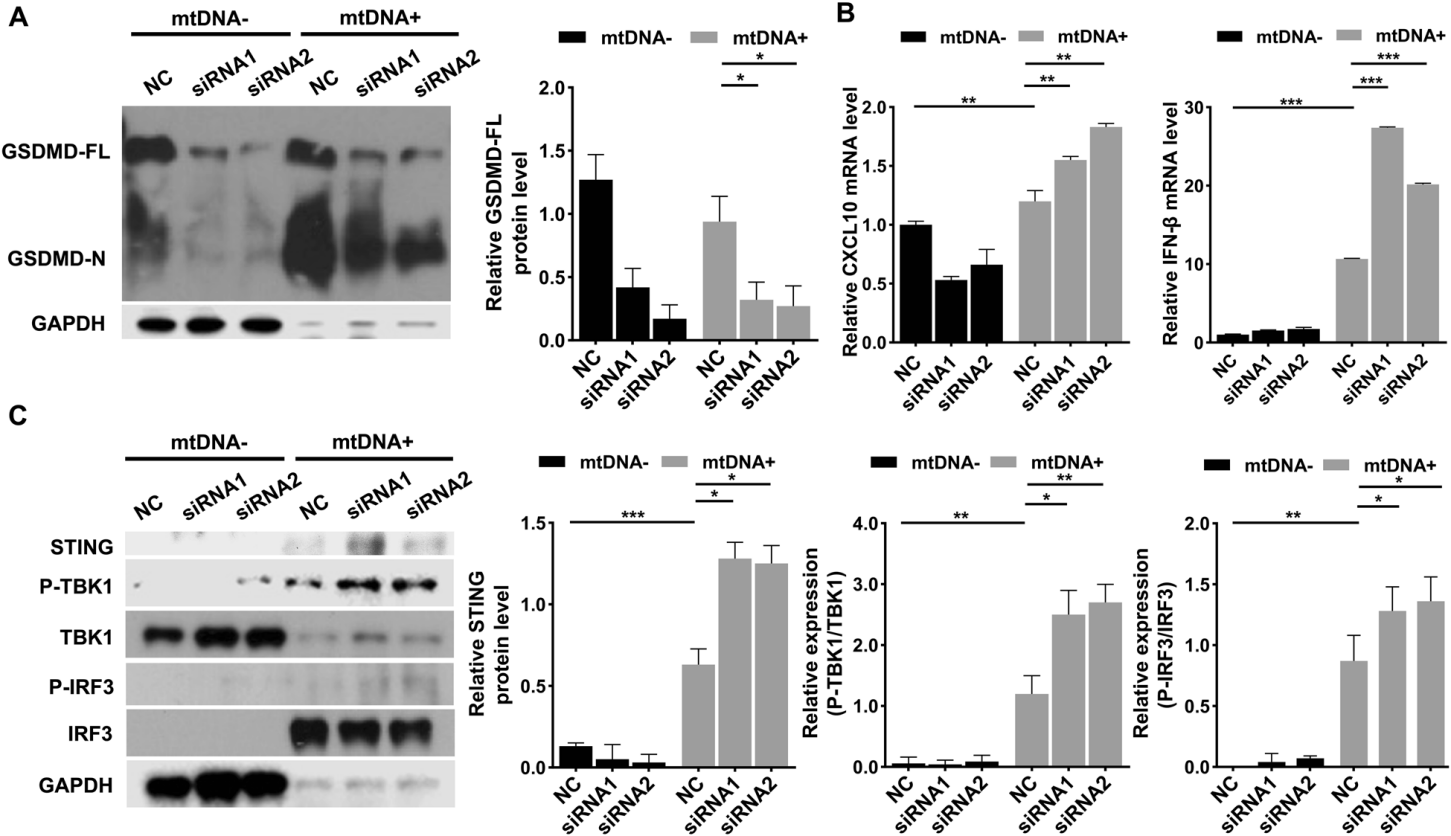
Cytosolic mtDNA activated the STING pathway after GSDMD knockdown. **A** In an interval 48 h, anti-GSDMD siRNA was transfected mDPC6T cells twice, after the second transfection mtDNA overexpressed. **B** The mRNA level of CXCL10 and IFN-β in mDPC6T cells was assessed by qPCR with or without GSDMD knockdown in response to 24 h of mtDNA transfection. **C** The protein expression of STING, P-TBK1/TBK1, P-IRF3/IRF3 was analyzed by western blotting with or without GSDMD knockdown in response to 24 h of mtDNA transfection. The data are the means ± standard deviations of 3 independent experiments (*p* < 0.05^*^, *p* < 0.01^**^, *p* < 0.001^***^).

To further investigate the specific effects of pyroptosis on odontoblast-like cell inflammation, we transfected mDPC6T cells with GSDMD-targeted small interfering RNA. Then, we achieved GSDMD silencing and performed western blotting to verify the transfection efficiency. The GSDMD knockdown efficiency was more than 80%, and the protein level of cleaved GSDMD was upregulated after transfection with mtDNA (Fig. 5A). To confirm the effect of GSDMD knockdown on odontoblast inflammation, inflammatory cytokines mRNA level was detected. Surprisingly, after mtDNA overexpression, the level of inflammatory cytokines, such as CXCL10 and IFN-β, was remarkably increased in GSDMD knockdown mDPC6T cells (Fig. 5B). Emerging research has revealed that the STING pathway activated by cytosolic mtDNA can be inhibited by GSDMD via regulation of K^+^ efflux, resulting in inhibition of the IFN-β response [16]. We hypothesized that GSDMD might inhibit the STING pathway in mDPC6T cells. Therefore, we analyzed the expression of the STING pathway and found that after mtDNA stimulation, the STING pathway was activated in odontoblast-like cells. Interestingly, the STING pathway was further upregulated in GSDMD knockdown mDPC6T cells overexpressing mtDNA (Fig. 5C). In short, overexpressed mtDNA in GSDMD deficiency cells, the STING pathway was activated which resulted in more severe odontoblast inflammation.

## Discussion

Pyroptosis is an inflammatory cell death caused by pathogen infection, which can trigger a strong inflammatory immune response [24]. Excessive pyroptosis has been confirmed to be involved in the pathogenesis of several inflammatory diseases, such as sepsis and infectious colitis [25, 26]. In the present study, we found that pyroptosis is closely associated with the progression of pulpitis. Moreover, we found that leaked cytosolic mtDNA acts as a DAMP to activate the NLRP3 inflammasome, causing pyroptosis and intensifying inflammation in odontoblast-like cells. From our previous [11] and present study, cytosolic mtDNA promotes pulp inflammation through the pyroptosis pathway and the STING pathway, suggesting that protecting mitochondria and reducing mtDNA leakage under inflammatory stimuli can delay the progression of inflammation. To further explore the relationship between these two signaling pathways, we found that the STING pathway was compensatorily activated after GSDMD knockdown. The innate immune response mediated by the STING pathway and cell death mediated by GSDMD are both involved in pulp inflammatory responses. Mechanistically, this study uncovered an unreported mechanism in inflamed odontoblast-like cells involving the mtDNA-GSDMD-STING pathway.

Emerging evidence has shown that overexpression of BAX can promote cell death by regulating the completeness of the outer mitochondrial membrane (OMM) [5, 27]. Inactive BAX exists in the cytoplasm, while activated BAX translocates to the OMM after inflammatory stimulation, which causes mitochondrial outer membrane permeabilization (MOMP), leading to the release of mitochondrial contents, such as mtDNA [5, 22]. The MOMP initiation signal cascade not only participates in many types of cell death but also induces proinflammatory signals to exert various nonlethal signaling functions [28]. Consistent with these studies, we found that inflammatory stimuli lead to mitochondrial damage. BAX from the cytoplasm accumulates in the mitochondrial membrane, and numerous DAMPs, such as mtDNA, are released via BAX, suggesting a potential connection between mtDNA and odontoblast inflammation. In melanoma, Cytochrome c released from damaged mitochondria activates caspase-3 to induce GSDME-mediated pyroptosis and participate in the tumor immune response [29]. However, whether any inflammatory stimuli other than mtDNA release from damaged mitochondria trigger the immune response of pulpitis still need to be further verified.

In this study, we first revealed that cytosolic mtDNA promotes pulpitis progression through GSDMD-mediated pyroptosis. GSDMD and the other indicators of the canonical pyroptosis pathway (NLRP3, cleaved caspase-1 and cleaved IL-1β) are elevated in inflammatory dental pulp. This indicates that cell pyroptosis occurs in human pulpitis tissue. Previous studies have indicated that the NLRP3 inflammasome can be activated by several types of stimuli, including K^+^ efflux, lysosomal disruption, calcium signaling, ER stress and mitochondrial alarmins (such as mtROS and mtDNA) [6, 30]. After overexpression of isolated mtDNA, typical pyroptosis morphologies, such as cell swelling, were observed under a light microscope, and western blotting showed upregulation of the pyroptosis executors GSDMD, NLRP3, cleaved caspase-1, and cleaved IL-1β in mDPC6T cells. Meanwhile, after EtBr treatment, the indicators of the canonical pyroptosis pathway were significantly suppressed, revealing that LPS may cause the canonical pyroptosis through mtDNA leakage in inflamed odontoblasts. However, as a special component of the outer wall of gram-negative bacteria, LPS can induce pyroptosis through canonical and noncanonical pathways. We will further study the role of other noncanonical pyroptosis pathways in pulpitis in the future.

The present study is the first to demonstrate that the STING pathway acts as a compensation for mtDNA-induced pyroptosis to increase pulp inflammation. We knocked down GSDMD to further explore the relationship between mtDNA and pyroptosis. When mtDNA was overexpressed, inflammatory cytokines, such CXCL10 and IFN-β, were increased in GSDMD knockdown cells, suggesting that STING activates IFN-β and CXCL10 production in the absence of GSDMD via mtDNA-associated PRRs and provide a compensatory mechanism for response to inflammatory infection in odontoblast GSDMD deficiency. Previous evidence has shown that mtDNA activates the STING pathway as a cell-intrinsic cGAS ligand and that cytosolic mtDNA mainly participates in the regulation of the cellular inflammatory response by activating the STING pathway [31, 32]. In macrophages, GSDMD can not only mediate pyroptosis but also inhibit activation of the STING pathway through K^+^ efflux to inhibit the IFN-β response [16, 17]. However, whether GSDMD has the same effect in odontoblasts remain curious. In our results, the phosphorylation of TBK1 and IRF3, downstream genes in the STING pathway, was increased in mDPC6T cells with GSDMD knockdown when mtDNA was overexpressed. The STING pathway was activated in odontoblasts overexpressing mtDNA when GSDMD was knocked down. The STING pathway, as the main signaling pathway for DNA sensing, has become known as a key mediator of inflammation due to environmental stimuli, such as infection and injury, and in conditions such as myocardial infarction, sepsis and systemic lupus erythematosus [33, 34]. Activation of the STING signaling may help prevent dangerous bacterial infection, and type I IFN may suppress adaptive immune responses, perhaps in an effort to control inflammation [35]. Nevertheless, the current research was only carried out in a cell model of gram-negative bacteria and lacks validation with gram-positive bacteria and in vivo data. The specific mechanism by which mtDNA participates in pyroptosis regulation requires further study. In future research, we aim to establish an in vivo model to verify these findings and conduct a more detailed study of the molecular mechanism.

**Fig. 6:**
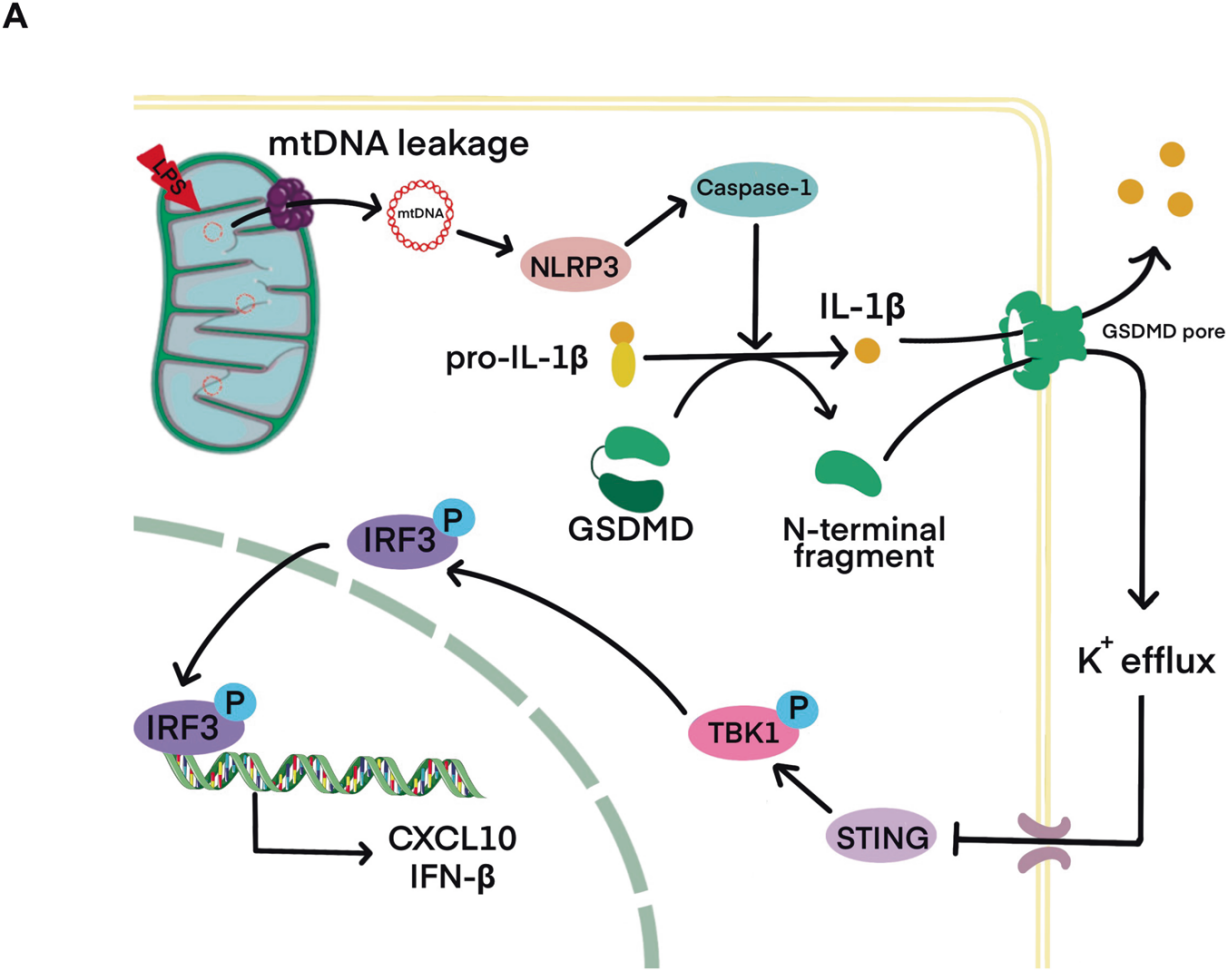
The mechanism diagram for cytosolic mitochondrial DNA regulates odontoblast inflammation. **A** Cytosolic mitochondrial DNA regulates odontoblast inflammation via pyroptosis. STING pathway compensatorily regulates the inflammatory process.

In conclusion, this study sheds light on the potential contribution of mtDNA-induced pyroptosis to pulpitis and provides a favorable target for pulpitis therapy. LPS-induced mitochondrial damage results in mtDNA leakage into the cytoplasm through activated BAX. Cytosolic mtDNA activates the NLRP3 inflammasome, thereby activating caspase-1 and GSDMD. The active N-terminal domain of GSDMD anchors to the cell membrane to form pores, causing cell IL-1β release and K ion outflow [16] and inhibiting the STING signaling pathway, thereby regulating the inflammatory response (Fig. 6A).

## Supplementary information

Appendix

## Data Availability

The data supporting the findings of this study are available from the corresponding author upon request.
