# Supplementary material for: Mitochondrial DNA leakage exacerbates odontoblast inflammation through gasdermin D-mediated pyroptosis
Source: Cell Death Discov. 2021 Dec 9;7:381. doi: 10.1038/s41420-021-00770-z (PMC8660913; doi:10.1038/s41420-021-00770-z)
Supplement: Supplementary file 1 — Appendix [file 41420_2021_770_MOESM1_ESM.docx]

**Appendix table 1**

**Antibodies**

| Antibodies | Source | Identifier |
| --- | --- | --- |
| Anti-NLRP3 | Abcam | ab263899 |
| Anti-pro caspase1+p10+p12 | Abcam | ab179515 |
| Anti-IL-1 beta | Abcam | ab234437 |
| Anti-GSDMD | Abcam | ab219800 |
| Anti-NAK/TBK1 | Abcam | ab40676 |
| Anti-IRF3 | Abcam | ab68481 |
| Anti-BAX | Proteintech | 60267 |
| STING | Cell Signaling  Technology | #13647 |
| Phospho-TBK1/NAK (Ser172) | Cell Signaling  Technology | #5483 |
| Phospho-IRF3  (Ser396) | Cell Signaling  Technology | #29047 |
| β-Tubulin | Bioprimacy | PMK059M |
| GAPDH | Abclonal | ac035 |

**Appendix table 2**

**Primer**

| Target | Primer sequence | |
| --- | --- | --- |
|  | Forward | Reverse |
| GSDMD | ATGCCATCGGCCTTTGAGAAA | AGGCTGTCCACCGGAATGA |
| CXCL10 | CCAAGTGCTGCCGTCATTTTC | TCCCTATGGCCCTCATTCTCA |
| IFN-β | CCTTCTTGATCTGCTGGG CA | TGGATGGCAAAGGCAGTGTA |
| tRNA-Leu^UUR^ | CTAGAAACCCCGAAACCAAA | CCAGCTATCACCAAGCTCGT |
| β2-Microglobulin | ATGGGAAGCCGAACATACTG | CAGTCTCAGTGGGGGTGAAT |
| GAPDH | TGTGTCCGTCGTGGATCTGA | TTGCTGTTGAAGTCGCAGGAG |

**Editing certification**
